# The Yule Approximation for the Site Frequency Spectrum after a Selective Sweep

**DOI:** 10.1371/journal.pone.0081738

**Published:** 2013-12-10

**Authors:** Sebastian Bossert, Peter Pfaffelhuber

**Affiliations:** Department of Mathematical Stochastics, Albert-Ludwigs University, Freiburg, Germany; University of Arkansas, United States of America

## Abstract

In the area of evolutionary theory, a key question is which portions of the genome of a species are targets of natural selection. Genetic hitchhiking is a theoretical concept that has helped to identify various such targets in natural populations. In the presence of recombination, a severe reduction in sequence diversity is expected around a strongly beneficial allele. The site frequency spectrum is an important tool in genome scans for selection and is composed of the numbers 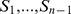, where 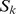 is the number of single nucleotide polymorphisms (SNPs) present in 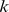 from 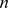 individuals. Previous work has shown that both the number of low- and high-frequency variants are elevated relative to neutral evolution when a strongly beneficial allele fixes. Here, we follow a recent investigation of genetic hitchhiking using a marked Yule process to obtain an analytical prediction of the site frequency spectrum in a panmictic population at the time of fixation of a highly beneficial mutation. We combine standard results from the neutral case with the effects of a selective sweep. As simulations show, the resulting formula produces predictions that are more accurate than previous approaches for the whole frequency spectrum. In particular, the formula correctly predicts the elevation of low- and high-frequency variants and is significantly more accurate than previously derived formulas for intermediate frequency variants.

## Introduction

Genetic hitchhiking is the cause of a severe reduction of sequence diversity in a population due to recent strong positive selection [1]. Several statistical methods are available to detect these selective sweeps. The most successful approaches include various aspects of the available data, such as the site frequency spectrum and linkage disequilibrium patterns. See e.g., [2] for a framework using a likelihood ratio test using the site frequency spectrum, [3], [4] for tests based on linkage disequilibrium and [5], who use a combination of both. The most challenging issue today is to dissect population demography from signatures of selection.

One of the most successful approaches for detecting selective sweeps is called *SweepFinder*. Here, the site frequency spectrum for a selective and a neutral model is compared for each SNP available in the data [6]. This approach highlights the necessity of making analytical predictions for site frequency spectra under strong positive selection, which is the main goal of the current manuscript. While *SweepFinder* uses a selective model with the star-like method (see e.g., [7]), here, we use a refined model.

Current theoretical investigations and predictions of the signature of strong positive selection are mostly based on a genealogical perspective. The resulting genealogy is termed coalescent in a random background and was studied by [8] and [9]. The simplest approximation for large selection coefficients is the star-like approximation from [10] and [7]. The star-like approximation assumes that all individuals from a sample taken at the time of fixation are direct descendants of the founder of the selective sweep. In addition, recombination events may have split the history of the target of selection from a linked neutral variant. [7], [11], and [12] used a marked Yule process, which has been shown to be a finer approximation by [7]. Rather than using a star-like approximation of the genealogy at the target of selection, [12] used the idea put forward by [13], which states that in the early phase of a selective sweep, the beneficial allele behaves similarly to a supercritical branching process. As a consequence, the genealogy also resembles a supercritical branching process, which turns out to be a Yule process [14].

In this manuscript, we go beyond approximating the genealogy by a marked Yule process and provide an analytical expression for the site frequency spectrum after a selective sweep. Two features of the spectrum are the most important for data analysis: an excess of singletons (which might also arise due to population expansion) and an excess of high-frequency variants (which appear to be a unique feature of sweeps; [15]). [16] already gave an approximation of the site frequency spectrum and used the excess of high frequency variants to develop a statistical test for positive selection. Using our analytical approximations, we will see that such classical approaches slightly overestimate the number of high-frequency variants, while our Yule-approximation is more accurate. In addition, intermediate-frequency variants are predicted accurately only by the marked Yule-approximation. These features of the Yule-approximation can be used to construct conservative tests for selective sweeps.

## Model and Results

Consider a (diploid) population of size 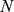 which evolves under the neutral Wright-Fisher model. We will study two loci (called 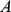
- and 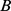-locus) within this population, which recombine with probability 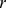 per generation. (We neglect recombination within loci.) At the 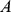-locus, the population is fixed for the wild-type 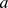 before time 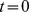. The 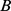-locus is modeled using an infinite sites model of mutation with mutation probability 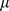 per generation (see [17]). At time 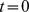, a beneficial mutation 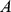 with fitness 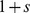 appears at the 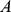-locus and is conditioned on eventual fixation in the whole population. Our main interest is the site frequency spectrum of the 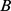-locus at the fixation time 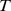 of the 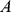-allele, which we also refer to as the end of the sweep. Consider a sample of size 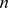 taken at time 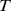, and let 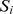 be the number of SNPs at the 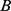-locus where the derived variant is present in exactly 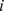 individuals. The time before 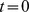 is called the *neutral phase*, while the time between 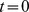 and 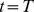 is the *selective phase*.

### Diffusion approximation and structured coalescent

**Figure 1:**
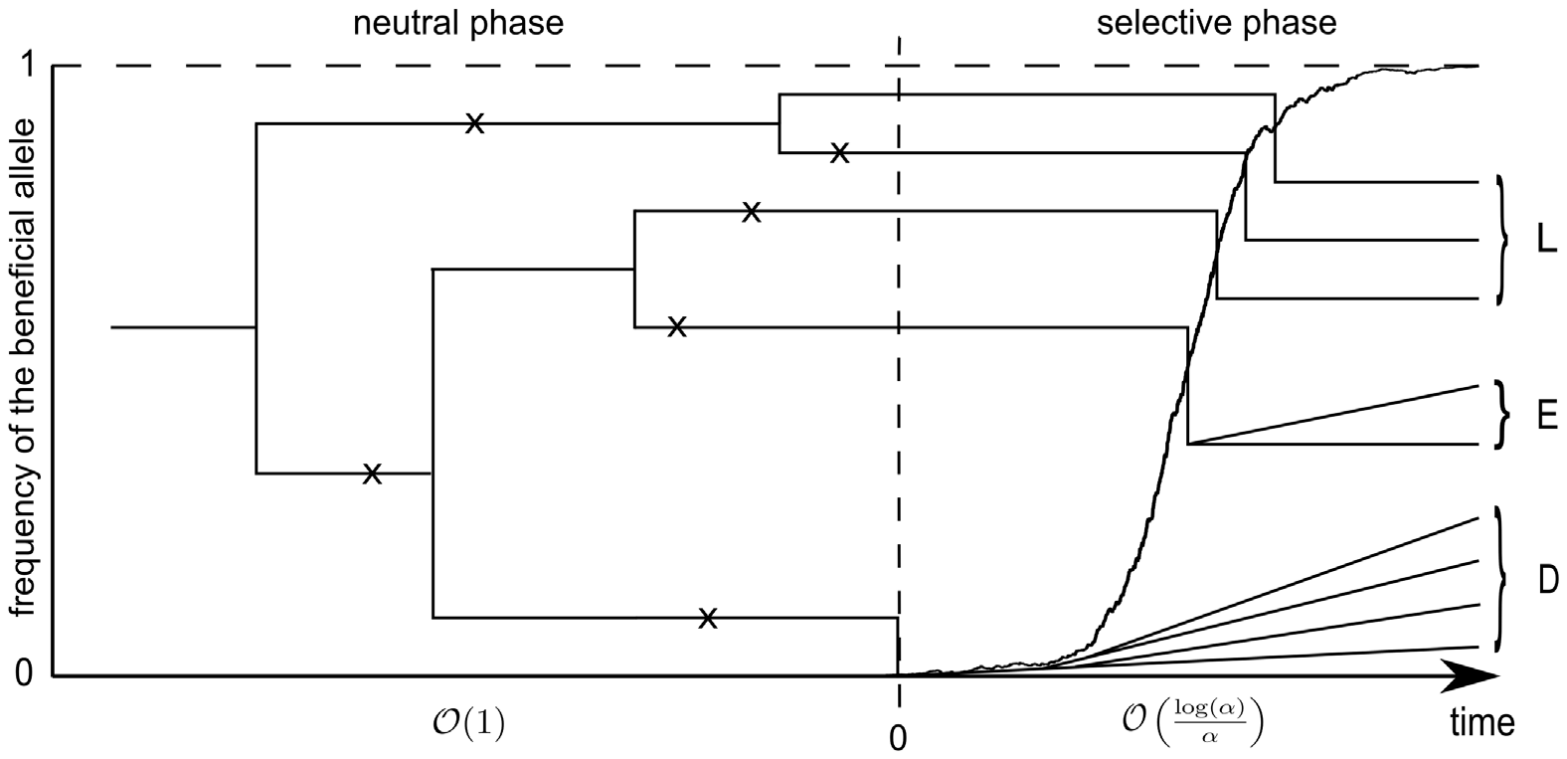
The structured coalescent. In the given example of the structured coalescent, we see on the right side the selective phase with a sample of size 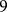 at the moment of fixation and the frequency development of the beneficial allele. At time 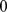, there are 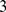 late recombinant families (labeled with 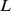), which all have a size of 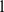, one early recombinant family (labeled with 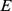) of size 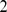 and one nonrecombinant family (labeled with 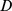) of size 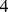. These lines then start a standard coalescent in the neutral phase. The crosses illustrate SNPs in the sample.

To derive an approximation of the expected site frequency spectrum, we rely on a diffusion approximation for the frequency of the beneficial 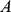-allele (see e.g., [18]) and a coalescent process in a random background as described in [9] (see also [8]). Recall (e.g., from [19]) that the frequency of the 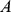-allele after 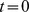, when time is rescaled by a factor of 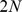, is approximately given by the solution 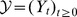 of the stochastic differential equation 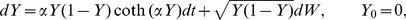
(1)where 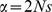 is the rescaled (genic) selection intensity, and 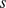 is defined by saying that 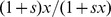 is the expected number of 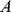-alleles in the next generation if the current frequency is 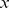. Observe that 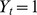 after some random time 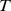, which we call the fixation time of 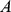. In the background of the path 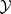, we consider a structured coalescent that evolves as follows (see Figure 1 for an illustration, where a sample of size 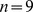 is used): Set 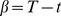 and start with 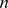 lines at time 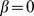 (i.e., 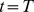 and the end of the sweep) in the 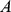-background. The following four transitions can occur between times 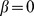 and 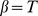, i.e., during the selective phase:

1. Coalescence of a pair of lines in the 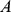-background: at rate 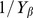, any pair of lines in the 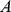-background coalesces.
2. Switching of background from 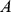 to 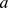 by recombination: at rate 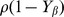 with 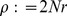 (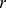 is the recombination fraction between the selective and neutral locus within a single generation), any line in the 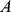-background changes to the 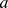-background.
3. Coalescence of a pair of lines in the 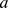-background: at rate 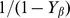, any pair of lines in the 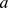-background coalesces.
4. Switching of background from 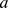 to 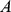 by recombination: at rate 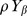, any line in the 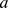-background changes to the 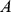-background.

Due to these transitions, there is a random number 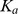 of lines in the 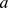-background at time 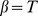 and 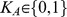 lines in the 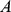-background. (If there was two or more lines in the 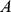-background, their coalescence rate would have been arbitrarily large by the coalescence rate 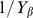.) The resulting 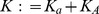 lines follow a standard neutral coalescent after time 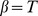, i.e., every pair of lines coalesces at rate 1 after only a single line is left and the process is stopped.

After having constructed the random tree from the coalescing lines, every line is hit by mutation events at the rate 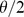, with 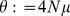. We call an event a *mutation of size* 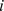 if it falls on a branch leading to exactly 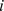 leaves of the tree. The number of size 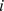 mutations is called 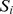, and 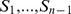 is called the site frequency spectrum, which we will approximate for large 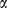 below.

### Yule approximation of the genealogy in the selective phase

In [19] and [11], the following approximation of the structured coalescent during the selective phase was developed with the limits of large 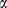 and for 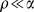: As was shown, events 3. and 4. from the structured coalescent can be ignored because their probability becomes small for large 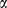. Thus, each line undergoes at most one recombination event during the selective phase. Two lines of the genealogy at time 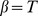 belong to the same family if they coalesce between time 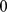 and 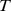. The following families are distinguished:

1. *Nonrecombinant family*: The set of individuals whose ancestral lineages never left background 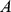.
2. *Early recombinant families*: The set of individuals whose ancestral lines have not left background 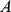 before (according to the backward time 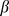) the first coalescence in the sample occurs, but the ancestor at time 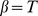 (equivalent to 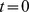) is in background 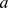.
3. *Late recombinant families:* The families consisting of a single individual whose ancestral line has left background 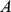 before the first coalescence in the sample, and the ancestor at time 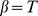 is in background 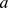.

Note that late recombinant families are of size 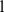 by definition, and there can be at most one nonrecombinant family that has inherited their 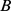-allele from the founder of the sweep.

To get an approximation formula for the genealogy at time 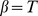, we first need the distribution for the number and size of the different families. Recall from Theorem 1 in [19] that the genealogy consists (up to an error of probability of order 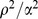) of

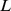
 *late recombinant* families of size 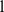,
- one *early recombinant* family of size 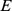 and
- one *nonrecombinant* family of size 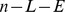.

For the joint distribution of 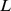 and 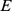, define a random variable 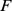, distributed according to 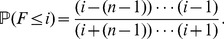
(2)


Given 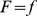, 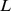 is a binomial random variable with 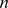 trials and success probability 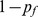, where



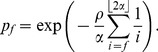
(3)


The distribution of 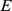 depends on 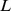 and on another variable 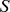, which gives the number of lines that are affected by the early recombination at time 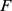 according to 
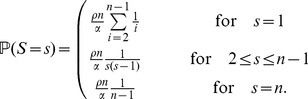
(4)(Note that the case 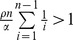 requires a different definition of the distribution of 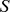, which we give in Section A of the SI.) As one or more of these 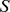 lines could experience a late recombination event, they could be kicked out of the family of early recombinants. This explains the hypergeometric distribution of 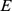, i.e., given 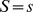 and 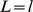, the variable 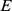 is hypergeometric with 
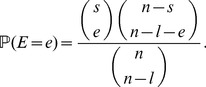
(5)


Combining these equations, a straightforward calculation (see Corollary 2.7 in [19]) leads to 
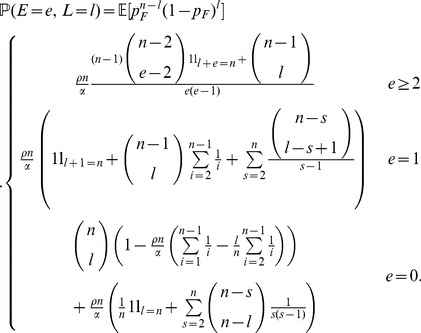
(6)


Note that this equation corrects an error (in the case of 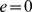) of the equation of [19]; see SI, Sections A and B. Moreover, there is a factor of 2 difference here because we assume a diffusion constant of 1 in (1).

### Yule approximation of the site frequency spectrum

Our goal is to obtain an expectation of the site frequency spectrum, 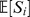, at the end of a selective sweep using the approximation from (6). We will assume that 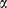 is large and that no new mutations accumulate in the sample during the selective phase. Moreover, recombination between the 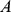
- and 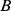-locus has to be in a certain range to see a non-trivial frequency spectrum. (Here, trivial would either mean that there is no variation at all if 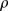 is too small or a neutral site frequency spectrum if 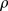 is too large.) Recalling that the duration of the sweep is approximately 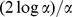 (see [19]), 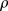 must be on the order of 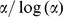. In other words, 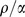 is on the order of 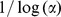 and hence small if 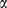 is large.

To get an approximation formula for the frequency spectra, the events and probabilities of the selective phase must be joined with the neutral phase. In the neutral phase, Kingman's coalescent describes the genealogy of the 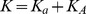 remaining lines. The crucial point is how to combine the approximation of the genealogy of the 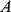-locus during the selective phase with a neutral coalescent before the onset of the sweep. A critical quantity is the number 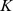 of ancestors of the sample at the onset of the sweep. Because a mutation can only influence at most 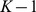 of these ancestors, the descendants in the selective phase depend on this number of lines. Recall that the sample size is 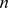, 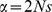 is large, 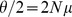 is the mutation rate and 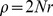 is the recombination rate, with 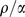 being small. Therefore, the expected number of mutations of size 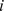 is (see SI, Section C for the proof) 
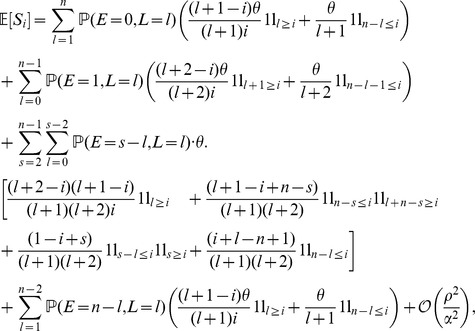
for 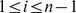, where the probabilities of 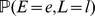 are given by (6). We note that the term 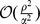 is due to the use of the approximation formula for the selective phase.

To get an idea of how this formula is computed, consider again Figure 1. There are 3 late recombinant families, one early recombinant family of size 2 and one nonrecombinant family (labeled 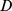) of size 4. Given these values, there are two different ways for a mutation to get to a size of 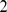. Either it had a size of 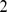 at time 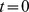 and these two lines were two late recombinant families, or it had size 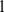 at time 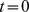 and then was the founder of the early recombinant family, which has a size of 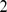 at the end of the sweep. Taking into account all possibilities, (7) arises.

### Previous approximation formulas

Using simulations, we compared the Yule approximation formula (7) to two other approximation formulas for the frequency spectra. The first approximation is from [16] and will be called the *deterministic formula* because a deterministic development of the frequency of allele 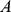 is assumed in this approach. The second approximation is the *star-like approximation* (see [7] or chapter 6 in [20]).

### Deterministic approximation

In [16], Fay and Wu obtained the following approximation for the site frequency spectrum after a selective sweep, building on the ideas of [1]. They obtain 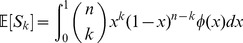
(8)with 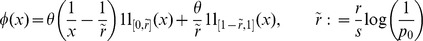
where 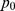 is the starting frequency of the beneficial allele. For the numerical comparison, we use 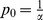 because, in this situation, the length of the selective phase is 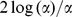, which is close to the expectation of the stochastic model.

### Star-like approximation

For the classical star-like approximation, every line in the selective phase has the same independent chance to recombine and be in background 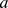 at time 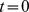. Therefore, 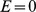, and 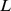 is binomially distributed with parameters 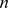 and 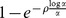, which is the probability that a single line recombines. Combining this insight with (7) leads to the equation 
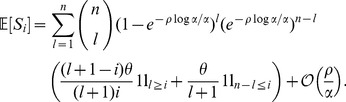
(9)


Note that for small 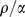, the approximation error is much larger than in (7).

### Numerical comparison

**Figure 2:**
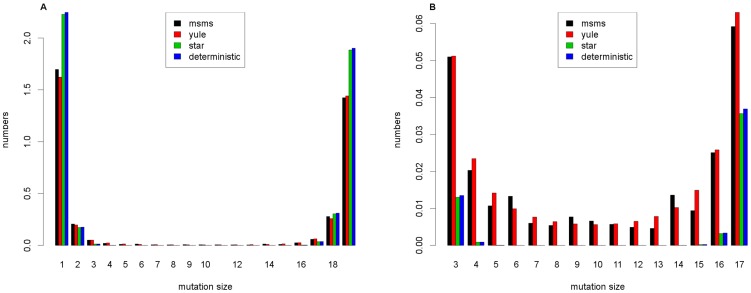
Comparison of the expected frequency spectra I. Comparison of the 3 approximation formulas and the results from msms for the parameters 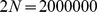, 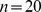, 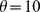, 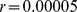 and 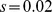. In **A**, the whole frequency spectrum is illustrated, while in **B**, the number of the mutation sizes between 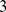 and 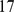 are enlarged.

Our goal is to compare the performance of the Formulas (7), (8) and (9) to simulations from the Wright-Fisher model. For the Wright-Fisher model, the simulation tool msms was used (which stands for *make sample mit selection*, see [21] or http://www.mabs.at/ewing/msms/index.shtml). To compare the different formulas for the expected frequency spectra, the average of 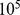 iterations was taken as a reference. Figure 2 shows the case of a high selective advantage 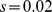 in a sample of size 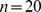. Theoretically, the Yule and star-like approximations converge for large 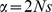. However, while the deterministic and star-like approximations perform about equally well, the (absolute and relative) error of (7) is smaller.

**Figure 3:**
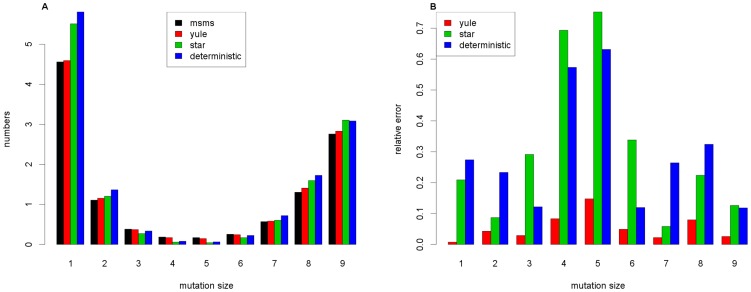
Comparison of the expected frequency spectra II. Comparison of the 3 approximation formulas and the results from msms for the parameters 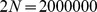, 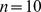, 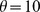, 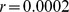 and 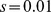. In **A**, we see the expected frequency spectra, and in **B**, we see the relative errors compared to the reference 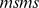.

In Figure 3, we used a smaller selective coefficient 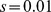 and a sample of size 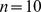. Here, the relative error of the star-like and deterministic approximations exceed 0.6. Again, the Yule approximation (7) gives the best results, with the relative error never exceeding 0.2. Reassuringly, all approximations give good results for low- and high-frequency variants that are known to be fundamental in detecting selecting sweeps in data.

**Figure 4:**
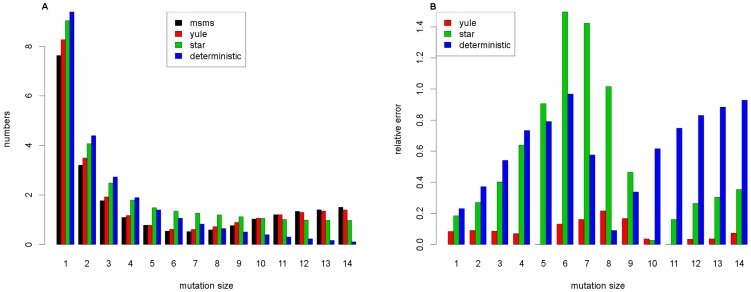
Comparison of the expected frequency spectra III. Comparison for the parameters 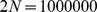, 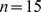, 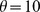, 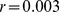 and 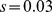, where the adjusted formula for the joint distribution according to Appendix A is needed. In **A**, the expected frequency spectra are depicted, and in **B**, the relative errors compared to the reference 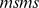 are illustrated.

In applications, the case of a high recombination rate is of particular importance. Here, (7) needs to be corrected as described in Appendix A. Because the error of all approximation formulas increases with recombination rate, it is no surprise that the errors in Figure 4 are larger than those in Figures 2 and 3. Still, the Yule approximation works best for most of the frequency classes.

## Discussion

The site frequency spectrum is a basic summary statistic used for the analysis of SNP data. Theoretical predictions of the shape of the frequency spectrum are most important in order to understand the evolutionary forces that have shaped the genomic data at hand. In the present paper, we have demonstrated how a recently developed approximation for selective sweeps from [7], [19], [11], [12], based on a marked Yule process, leads to such a prediction (at least for the expected site frequency spectrum). For the analytical formula, two cases have to be taken into account. If 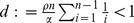, the marked Yule process can be applied directly, but if 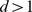, we have to use some normalization procedure. The latter case arises if the neutral locus has a large recombinational distance to the target of selection. In the parameter constellation of Figure 4, neither of the approximations works particularly well, with relative errors up to 20% for the Yule and deterministic approximations and over 140% for the star-like approximation. However, theoretical predictions become worse for larger 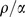 and errors are less predicable in this setting.

For smaller recombinational distances, we find that the Yule approximation outperforms the star-like approximation, especially for intermediate frequency variants (relative error up to 20% for the Yule approximation versus up to 80% for the star-like approximation, see Figure 3). In a comparison between the Yule and star-like approximations, a basic difference is that the star-like approximation forbids what we called *early recombinant families*. Such families lead to a decrease in the number of singleton mutations, which is shown in our simulations and has the greatest impact on the relative errors we reported above.

Altogether, the combination of (7) and (11) gives our analytical formula. Most importantly, compared to other approaches, such as the deterministic approach of [16] and the star-like approximation derived in [10], [7] and used e.g., in [3], the Yule process approximation has a smaller error in nearly all cases. Although the formulas derived in the Yule approximation are more involved, they can still be easily implemented for data applications to obtain a higher accuracy. Above all, such accuracy is desirable in genome scans for selective sweeps, which are frequently carried out by software such as *SweepFinder* [6].

## Supporting Information

Appendix S1 Supporting Information for the article.
(PDF)
