# Correction: The Yule Approximation for the Site Frequency Spectrum after a Selective Sweep

**DOI:** 10.1371/annotation/48c6407a-b902-47ef-b57b-5bdca48b9991

**Published:** 2014-01-22

**Authors:** Sebastian Bossert, Peter Pfaffelhuber

This article was republished on January 17th, 2014 to include a document containing the Supporting Information for the article that was incorrectly omitted from the original edition of the article. The original edition of the article is available here: http://plosone.org/corrections/pone.0081738.original.cn.pdf

