# Supplementary material for: The Yule Approximation for the Site Frequency Spectrum after a Selective Sweep
Source: PLoS One. 2013 Dec 10;8(12):e81738. doi: 10.1371/journal.pone.0081738 (PMC3858263; doi:10.1371/journal.pone.0081738)
Supplement: Appendix S1 — Supporting Information for the article. (PDF) [file pone.0081738.s001.pdf]

The Yule approximation for the site frequency spectrum after a selective sweep, Supporting Information

Sebastian Bossert, Peter Pfaffelhuber

November 7, 2013

Section A

In the case of $\frac{\rho n}{\alpha}\sum_{i=1}^{n-1}\frac{1}{i} > 1$ the values of equation (4) would lead to a negative probability for the case of $S = 0$. To avoid this occurrence, we have to rescale the different values by $m := \max\left(1, \frac{\rho n}{\alpha}\sum_{i=1}^{n-1}\frac{1}{i}\right)$. Thus, the full definition of the distribution of S is given by

$$\mathbb{P}(S = s) = \begin{cases} \frac{\rho n}{\alpha m}\sum_{i=2}^{n-1}\frac{1}{i} & \text{for} \quad s = 1 \\ \frac{\rho n}{\alpha m(s(s-1))} & \text{for} \quad 2 \le s \le n-1 \\ \frac{\rho n}{\alpha m(n-1)} & \text{for} \quad s = n. \end{cases} \tag{10}$$

Using this definition to formulate the joint distribution of E and L leads to

$$\begin{aligned}\mathbb{P}(E = e, L = l) &= \mathbb{E}[p_F^{n-l}(1-p_F)^l] \\ &\cdot \begin{cases} \frac{\rho n}{\alpha m}\frac{(n-1)\binom{n-2}{e-2}\mathbb{1}_{l+e=n} + \binom{n-1}{l}}{e(e-1)} & e \ge 2 \\ \frac{\rho n}{\alpha m}\left(\mathbb{1}_{l+1=n} + \binom{n-1}{l}\sum_{i=2}^{n-1}\frac{1}{i} + \sum_{s=2}^{n}\frac{\binom{n-s}{l-s+1}}{s-1}\right) & e = 1 \\ \max\left(0, \binom{n}{l}\left(1 - \frac{\rho n}{\alpha}\sum_{i=1}^{n-1}\frac{1}{i}\right)\right) & \\ \quad + \frac{\rho n}{\alpha m}\left(\frac{1}{n}\mathbb{1}_{l=n} + \binom{n-1}{l-1}\sum_{i=2}^{n-1}\frac{1}{i} + \sum_{s=2}^{n}\binom{n-s}{n-l}\frac{1}{s(s-1)}\right) & e = 0. \end{cases}\end{aligned} \tag{11}$$

Section B

Corrected proof for the calculation of $\mathbb{P}(E=0, L=l)$ (see Corollary 2.7 in [1])
The probability for $E=0$ and $L=l$ can first be expressed by

$$\mathbb{P}(E=0, L=l) = \mathbb{E}[p_F^{n-l}(1-p_F)^l] \underbrace{\sum_{s=0}^{n} \binom{n-s}{n-l} \mathbb{P}(S=s)}_{=:u}. \tag{12}$$

Now, we can calculate u by using (4)

$$\begin{aligned} u &= \binom{n}{l}\left(1 - \frac{n\rho}{\alpha}\sum_{i=1}^{n-1}\frac{1}{i}\right) \\ &\quad + \frac{n\rho}{\alpha}\left(\frac{1}{n}\mathbb{1}_{l=n} + \binom{n-1}{l-1}\sum_{i=2}^{n-1}\frac{1}{i} + \sum_{s=2}^{n}\binom{n-s}{n-l}\frac{1}{s(s-1)}\right) \\ &= \binom{n}{l}\left(1 - \frac{n\rho}{\alpha}\left(\sum_{i=1}^{n-1}\frac{1}{i} - \frac{l}{n}\sum_{i=2}^{n-1}\frac{1}{i}\right)\right) \\ &\quad + \frac{n\rho}{\alpha}\left(\frac{1}{n}\mathbb{1}_{l=n} + \sum_{s=2}^{n}\binom{n-s}{n-l}\frac{1}{s(s-1)}\right). \end{aligned} \tag{13}$$

Section C

Proof of (7)
By our assumptions, new mutations hit the genealogy of the sample only in the neutral phase. Therefore, we have to combine our understanding from the selective phase as given by the Yule approximation of the genealogy with the neutral site frequency spectrum. The expected frequency spectra at time $t=0$ of a sample of size k, i.e., after the neutral phase, is given by

$$\mathbb{E}[S_i] = \frac{\theta}{i}, \qquad 0 < i < k. \tag{14}$$

In our case, the sample size k at time $t=0$ depends on the genealogy in the selective phase. We combined the probability for different genealogies in the selective phase with the corresponding expected frequency spectra. We will distinguish 3 different situations:

a) There is no early recombinant family, i.e., $E=0$.

b) There is an early recombinant family, but it consists of only one individual, i.e., $E=1$.

c) There is an early recombinant family, and it consists of more then one individual, i.e., $E \geq 2$.

The different situations are displayed in Figure 5 for a sample of size 5. Additionally, for every situation, different cases must be distinguished. Here, we present the situations $E=0$ and $E \geq 2$

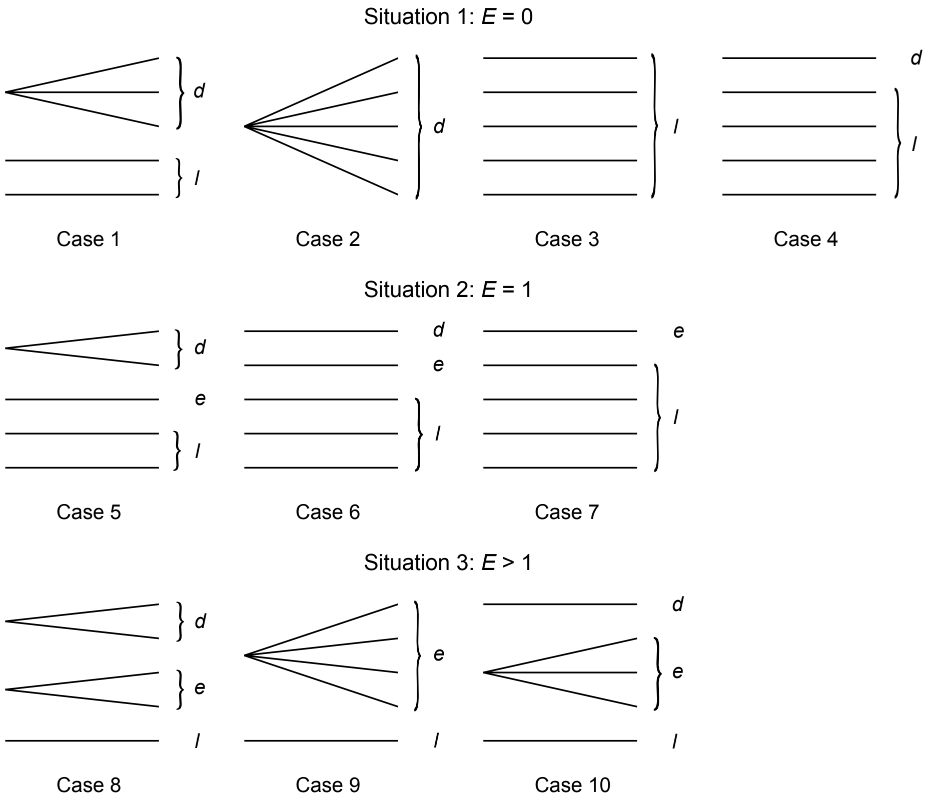

Figure 5: Here, the different situations for a sample of size 5 are pictured. In the course of the proof for every situation, diverse cases must be distinguished because they need different approaches.

because the situation $E = 1$ can be solved analogously. First, we set D as the number of lines in the sample that go back to the founder of the sweep; hence, $D := n - E - L$.

For $E = 0$, cases 2, 3 and 4 from Figure 5 are easy to handle. If $L = 0$, then all individuals coalesce during the selective phase and no mutations are in the sample, so

$$\mathbb{E}[S_i \mid E = 0, L = 0] = 0, \qquad 0 < i < n. \tag{15}$$

If $L = n$ or $L = n - 1$, then the number of lines before and after the selective phase are the same, i.e., $K = n$. Therefore, only the neutral phase has an effect on the frequency spectra, which leads to

$$\mathbb{E}[S_i \mid E = 0, L = n] = \mathbb{E}[S_i \mid E = 0, L = n - 1] = \frac{\theta}{i}. \tag{16}$$

In the case that $l \in \{1, \dots n - 2\}$, note that there are $l + 1$ lines at the end of the neutral phase. We must consider two different events, which lead to a mutation of size i. Either the mutation achieves a size of i before the selective phase and none of these lines split during the selective phase or the mutation has size $i - d + 1$ before the selective phase and one of these lines is hit by the founder of the sweep.

In the first event, the mutation affects i lines, leading to $\binom{l+1}{i}$ possibilities to select the i lines that have the mutation. The line that splits must not be one of the i lines, so the probability that a mutation of size i keeps its size is $\frac{\binom{l}{i}}{\binom{l+1}{i}}$. Combining this probability with the expected number of mutations under neutrality – see (14) – gives the expected number of mutations that achieve a size of i by this first event

$$\frac{\theta}{i}\frac{\binom{l}{i}}{\binom{l+1}{i}}\mathbb{1}_{l\geq i} = \frac{\theta}{i}\frac{\frac{l!}{i!(l-i)!}}{\frac{(l+1)!}{i!(l+1-i)!}}\mathbb{1}_{l\geq i} = \frac{(l+1-i)\theta}{(l+1)i}\mathbb{1}_{l\geq i}. \tag{17}$$

Using the same considerations for the second event (size i is not reached until the end of the selective phase), the conditional expectation in the case $l \in \{1, \dots n - 2\}$ is obtained by

$$\mathbb{E}[S_i \mid E = 0, L = l] = \frac{(l+1-i)\theta}{(l+1)i}\mathbb{1}_{l\geq i} + \frac{\theta}{l+1}\mathbb{1}_{d\leq i}. \tag{18}$$

For $l \in \{n - 1, n\}$, we use the equation

$$\frac{(l+1-i)\theta}{(l+1)i} + \frac{\theta}{l+1} = \frac{\theta}{i} \tag{19}$$

and the fact that $\mathrm{l}_{l\geq i}$ and $\mathrm{l}_{d\leq i}$ is always true for $l = n$ and $l = n - 1$.

In the situation $E > 1$, cases 9 ($D = 0$) and 10 ($D = 1$) (see Figure 5) can be handled in the same way as before. There is only one line that splits (now e and not d) in the selective phase, such that a mutation again has two possibilities to get to a size of i. Therefore, let us concentrate on the more challenging case 8, $E > 1$ and $D > 1$. Note that in this case, we have $K = l + 2$ lines, which are present at the beginning of the selective phase. There are a total of four possibilities that cause a mutation of size i. There are two coalescence events that must be considered, one for the early recombinants and one for the lines going to the founder of the sweep (see Figure 5). We distinguish the following four events:

- **event** 1**:** a mutation grows before the selective phase to a size of i and none of these lines is hit by the two lines that go back to the founder or the early recombinant family. (This event is only possible for $l \geq i$.)
- **event** 2**:** a mutation grows to a size of $i-d+1$ before the selective phase and one of these lines is hit by the founder, but no line is hit by the early recombinant family. (This event is only possible for $d \leq i$ and $d+l \geq i$.)
- **event** 3**:** a mutation grows to a size of $i-e+1$ before the selective phase and one of these lines is hit by the early recombinant family, but no line is hit by the founder. (This alternative is only possible for $e \leq i$ and $e+l \geq i$.)
- **event** 4**:** a mutation grows to a size of $i-e-d+2$ before the selective phase, one of these lines is hit by the founder and another line is hit by the early recombinant family. (This alternative is only possible for $e+d \leq i$.)

Each event can be treated similar to situation 1, except for the fact that there are now 2 drawings. We will explain event 2 to show the procedure:

At the beginning of the selective phase, the mutation has size $i-d+1$, so there are $\binom{l+2}{i-d+1}$ possibilities to distribute the mutation among the lines and $\binom{1}{1}\binom{l}{i-d}$ possibilities to receive the designated result because one line must be hit by the founder of the sweep and the other $i-d$ lines can be distributed among l lines. Combining the arising probability with the expected frequency spectra before the selective phase leads to

$$\frac{\theta}{(i-d+1)}\frac{\binom{l}{i-d}}{\binom{l+2}{i-d+1}} = \frac{\theta(l+1-i+d)}{(l+2)(l+1)}\mathbb{1}_{d\leq i}\mathbb{1}_{d+l\geq i} \tag{20}$$

for the expected number of mutations that achieve a size of i due to event 2.

In total, under the condition $e+l \in \{2,\dots n-2\}$ (because in case 8 we have $D \geq 2$) and $L=l$, we get the equation

$$\begin{aligned}\mathbb{E}[S_i \mid E=e, L=l] = {}& \frac{\theta(l+2-i)(l+1-i)}{i(l+2)(l+1)}\mathbb{1}_{l\geq i} + \frac{\theta(l+1-i+d)}{(l+2)(l+1)}\mathbb{1}_{d\leq i}\mathbb{1}_{d+l\geq i} \\ &+ \frac{\theta(e+l+1-i)}{(l+2)(l+1)}\mathbb{1}_{e\leq i}\mathbb{1}_{e+l\geq i} + \frac{\theta(i+l-n+1)}{(l+2)(l+1)}\mathbb{1}_{e+d\leq i}.\end{aligned} \tag{21}$$

Now, we want to use the conditioned expectations to obtain the expectation $\mathbb{E}[S_i]$. For this purpose, we split the expectation on conditioned expectations, that is,

$$\begin{aligned}\mathbb{E}[S_i] = {}& \sum_{l=1}^{n}\mathbb{P}(E=0,L=l)\mathbb{E}[S_i \mid E=0,L=l] + \sum_{l=0}^{n-1}\mathbb{P}(E=1,L=l)\mathbb{E}[S_i \mid E=1,L=l] \\ &+ \sum_{s=2}^{n-1}\sum_{l=0}^{s-2}\mathbb{P}(E=s-l,L=l)\mathbb{E}[S_i \mid E=s-l,L=l] \\ &+ \sum_{l=1}^{n-2}\mathbb{P}(E=n-l,L=l)\mathbb{E}[S_i \mid E=n-l,L=l] + \mathcal{O}\left(\frac{\rho^2}{\alpha^2}\right),\end{aligned} \tag{22}$$

where the $\mathcal{O}$ term appears because of the approximation character of the applied formula for the selective phase. Now, we can insert the conditioned expectations to obtain the final result

$$\begin{aligned}
\mathbb{E}[S_i] &= \sum_{l=1}^{n} \mathbb{P}(E=0, L=l)\left(\frac{(l+1-i)\theta}{(l+1)i}\mathbb{1}_{l\geq i} + \frac{\theta}{l+1}\mathbb{1}_{n-l\leq i}\right) \\
&+ \sum_{l=0}^{n-1} \mathbb{P}(E=1, L=l)\left(\frac{(l+2-i)\theta}{(l+2)i}\mathbb{1}_{l+1\geq i} + \frac{\theta}{l+2}\mathbb{1}_{n-l-1\leq i}\right) \\
&+ \sum_{s=2}^{n-1}\sum_{l=0}^{s-2} \mathbb{P}(E=s-l, L=l)\cdot\theta \\
&\qquad \cdot\left[\frac{(l+2-i)(l+1-i)}{(l+1)(l+2)i}\mathbb{1}_{l\geq i} + \frac{(l+1-i+n-s)}{(l+1)(l+2)}\mathbb{1}_{n-s\leq i}\mathbb{1}_{l+n-s\geq i}\right. \\
&\qquad\qquad \left. + \frac{(1-i+s)}{(l+1)(l+2)}\mathbb{1}_{s-l\leq i}\mathbb{1}_{s\geq i} + \frac{(i+l-n+1)}{(l+1)(l+2)}\mathbb{1}_{n-l\leq i}\right] \\
&+ \sum_{l=1}^{n-2} \mathbb{P}(E=n-l, L=l)\left(\frac{(l+1-i)\theta}{(l+1)i}\mathbb{1}_{l\geq i} + \frac{\theta}{l+1}\mathbb{1}_{n-l\leq i}\right) + \mathcal{O}\left(\frac{\rho^2}{\alpha^2}\right).
\end{aligned}$$

References

[1] Etheridge A, Pfaffelhuber P, Wakolbinger A (2006) An approximate sampling formula under genetic hitchhiking. Ann Appl Probab 16: 685–729.
